# Does reactivation of cytomegalovirus contribute to severe COVID-19 disease?

**DOI:** 10.1186/s12979-021-00218-z

**Published:** 2021-03-12

**Authors:** Cecilia Söderberg-Nauclér

**Affiliations:** Department of Medicine, Solna, Microbial Pathogenesis Unit, Karolinska Institutet, and Division of Neurology, Karolinska University Hospital, Bioclinicum, Visionsgatan 4, 171 64 Solna, Stockholm, Sweden

## Abstract

The majority of people infected with SARS-CoV-2 are asymptomatic or have mild to moderate symptoms. However, for unknown reasons, about 15 % have severe pneumonia requiring hospital care and oxygen support, and about 5 % develop acute respiratory distress syndrome, septic shock, and multiorgan failure that result in a high mortality rate. The risk of severe COVID-19 is highest among those who are over 70 years of age. Why severe COVID-19 develops in some people but not others is not understood. Could some cases involve reactivation of latent cytomegalovirus (CMV)?

## Key points

Latent human cytomegalovirus (CMV) is carried by 70–90 % of the adult population and is reactivated by inflammation. One third of patients in intensive care reactivate CMV, which doubles their mortality rate; how many COVID-19 patients reactivate latent CMV to complicate their diseases and enhance their mortality rate?

## Background

On January 3 2020, Chinese officials provided information to WHO on the cluster of cases of ‘viral pneumonia of unknown cause’ discovered in Wuhan. The virus causing this pneumonia was identified as SARS-CoV-2, spread fast and rapidly enhanced healthcare needs for patients requiring oxygen support and respirators, leading to increasing death tolls. Following the alarming increase of cases worldwide, the WHO declared COVID-19 as a pandemic on March 11, 2020. One year later, in January 2021 over 100 million people had been confirmed to be infected with SARS-CoV-2 and over 2.16 million people had died of COVID-19 disease world-wide, making this pandemic the most challenging since the Spanish flu in 1918.

### Who becomes severely ill in COVID-19 disease?

The virus causes asymptomatic, mild and severe infections. While many SARS-CoV-2 infected individuals are asymptomatic (estimated to account for 40–50 % of transmissions) and a majority of infected individuals develop mild to moderate symptoms, about 15 % have severe pneumonia requiring hospital care and oxygen support, and about 5 % develop acute respiratory distress syndrome, septic shock, and multiorgan failure that result in a high mortality rate [1, 2]. The severity of the disease and the mortality risk increases with age, but previously healthy young persons may also develop severe disease and need intensive care unit (ICU) treatment.

The main known risk factors for severe COVID-19 are high blood pressure, obesity, type II diabetes, cardiovascular diseases, male sex and high age [1, 2]. The overall estimated death rates range from 0.3 to 1.0 %, but are strikingly higher in those over 70, and especially in those above 80 years of age, reaching up to 5–16 % with highest risk in men [3, 4]. The reason for this is not known, but it is plausible that the well-known dysregulation of immune functions in the elderly (“immune senescence”) could contribute to an inability to combat the new pathogen SARS-CoV-2 and also give rise to a dysregulated immune response. In 2002, Wikby and colleagues identified cytomegalovirus (CMV) as a key component of the immune risk profile in the elderly, which was associated with increased mortality within 2 years [5]. Of note, the influence of CMV on health seems to be less significant in women than men [6], and men are worse affected by SARS-CoV-2. Later studies confirmed these original observations [7, 8] and described an increasing accumulation of CMV specific memory and effector T cells with increasing age. This phenomenon is also apparent in murine CMV studies and is believed to be caused by a repeated exposure of antigens from the pathogen [9], and imply that the inflation of the immune system is caused by a chronic presentation of CMV peptides to the immune system.

Under these circumstances, the immune system becomes busy keeping CMV at bay, which leads to an expansion of the T cells pool that is directed towards CMV. At cost, the naive T cell pool decreases and makes it more difficult for older CMV positive individuals to generate an adaptive immune response to combat new infections, such as the novel SARS-CoV-2 virus. Naïve T cells are recruited from the thymus, which is highly active in newborns, but 99 % reduced in people over 70 years of age. It has been estimated that CMV drives the attrition of the naive T cells pool by about 20 years [10]. The impaired immunity in CMV positive elderly individuals can also be caused by impaired cellular functions [11]. An active CMV infection will lead to immunosuppression via direct inhibitory effects on antigen presentation, NK, B and T cells responses, via sophisticated viral immune evasion strategies[12]. As a consequence, CMV associated immune senescence and immunosuppression in the elderly may increase their risk of dying from influenza and other infectious diseases [13], like SARS-CoV-2.

Considering the above circumstances, a vicious cycle may be generated, in which it would be difficult to determine what was the hen or the egg in this condition in relation to CMV. Will elderly with an impaired and exhausted immune response reactivate CMV and maintain a low-grade chronic infection, which further, through its negative effects of immune cells, impair the outcome of a SARS-CoV-2 infection? This is difficult to separate from a scenario in which SARS-CoV-2 infection itself, through mechanisms that drive reactivation, and the reactivated virus´ ability to cause hyper inflammation and simultaneous immunosuppression leading to worsened clinical outcome, as was recently discussed by Moss [14] and Kadambari et al. [15].

It is interesting to note that CMV has been associated with several diseases that increase risk for severe COVID-19, as well as with thrombotic events [16–18], which are main complications of COVID-19 disease. CMV is described in several case reports of atypical Kawasaki disease in children [19], in some aspects resembling the rare observed COVID-19 associated multisystem inflammatory syndrome in children (MIS-C) occurring 3–6 weeks after a SARS-CoV-2 infection [20]. Is it possible that CMV is reactivated in some patients who develop severe COVID-19, contributing to its pathogenesis and high mortality rate? I will here discuss mechanistic aspects of CMV and SARS-CoV-2 interactions in humans.

## Main text

CMV is a herpesvirus that generally infects small children. It causes congenital infection in 0.5–2.2 % of infants and is transmitted through breast milk. By 1 year of age, 30–40 % of children are seropositive for CMV [21]. The virus spreads in the population through close personal contact and via sexual transmission; by adulthood, 60–100 % of people carry the virus [22, 23]. The CMV seroprevalence varies depending on the country’s socioeconomic status. It is approaching 100 % in Low-to-Middle-Income Countries (LMIC); people living in these countries generally have a lower life expectancy, and they are less affected by severe COVID-19 mortality. In contrast, WEIRD countries (Western, educated, industrialized, rich and democratic) have higher life expectancy; and their elderly population is the most vulnerable to severe COVID-19 disease. This may explain why African countries with a younger population are not as hard hit by COVID-19 death tolls as European countries and the US. Among Americans, black people are among the most highly affected ethnic group by severe COVID-19. In the US, the seroprevalence of CMV was 76 % and 82 % among non-Hispanic black and Mexican American groups, but 51 % in non-Hispanic white people [24]. Similar observations were made 20 years ago in the UK, where 46 % of white pregnant women were CMV positive, as compared with 77 % of Afro/Caribbean and 88 % of Asian women, respectively [25]. For many years, CMV infection was not considered to cause any pathology in healthy people. The primary infection produces mild or no clinical symptoms but results in a life-long latent or persistent infection, from which reactivation may occur throughout life [22]. However, emerging evidence suggests that CMV is linked to cancer and chronic inflammatory diseases [26, 27], including those associated with increased risk of COVID-19.

CMV reactivation is driven by immune activation and inflammation [28, 29]. In organ transplant patients, the allogeneic immune reaction causes rejection and leads to CMV reactivation [30]. In the absence of prophylactic treatment, CMV is reactivated in 40–80 % of transplant recipients who may develop viremia or organ invasive disease [31]. Their immunosuppression poses them at risk of developing clinical CMV disease. The lungs are a major reservoir for latent virus [32]. Lung transplant patients may develop life-threatening CMV pneumonia, mainly through immunopathologic mechanisms, which is often followed by bronchiolitis obliterans, lung fibrosis, and poor lung function (chronic rejection in the lung graft) [33, 34]. CMV is often reactivated in heart transplant patients and can increase the risk of transplant vasculopathy and fibrosis, leading to poor heart function or occlusion of the blood supply to the heart [35–37]. CMV is also linked to posttransplant diabetes [38]. These complications are reduced in patients who receive antiviral prophylactics against CMV [39, 40].

In immunocompetent people, CMV infection is associated with hypertension, deep vein thrombosis, diabetes, myocardial infarction, and stroke [41–43]. CMV proteins are detected in the vasculature in the absence of viremia, and represent one example of chronic CMV antigen stimulation of immune cells that could drive inflation. Moss recently discussed that CMV could represent a potential cofactor in metabolic and cardiovascular complications in COVID-19 patients [14]. In sepsis patients, a strong inflammatory reaction may reactivate the virus within 4–7 days [44]—about the same time required for CMV reactivation *in vitro* [45]. Thus, SARS-CoV-2 infection and its strong activation of the innate immune system may trigger CMV reactivation in organs such as the lungs and bowel of COVID-19 patients. Notably, most COVID-19 patients who end up in the ICU arrive there about 8–11 days after they become sick [1]. CMV is reactivated in 30–35 % patients under ICU care and doubles their mortality rate [46]; however, it may take several weeks for patients to become viremic. A higher viral load of CMV is associated with enhanced mortality, and randomised trials show a lower reactivation rate in patients who receive antiviral prophylaxis for CMV [47]. If CMV is reactivated in COVID-19 patients and develops as a co-infection with SARS-CoV-2, the two viruses may have detrimental effects. They would be expected to suppress or even kill T cells and natural killer cells; activate macrophages and neutrophils in a cascade of events leading to a point of no return from inflammation; and could then affect endothelial cells and thrombocytes to cause coagulopathy and thrombus formation—precisely as observed in COVID-19 patients.

COVID-19 is characterized by pneumonia, lymphopenia, exhausted T cells, and a cytokine storm with high levels of interleukin (IL)-1β, IL-2, IL-2R, IL-6, IL-8, IL-10, IL-17, granulocyte colony-stimulating factor (G-CSF), granulocyte-macrophage CSF, and tumor necrosis factor (TNF)-α and high levels of chemokines such as monocyte chemoattractant protein (MCP)-1, macrophage inflammatory protein (MIP)-1α, and IFN-inducing protein (IP) 10 [2, 48]—all of which are produced during CMV infections [16, 49]. The massive infiltration of macrophages into the lungs may be mediated by MCP-1, MIP-1α, and IP10 produced by infected epithelial cells. Many of the infiltrating macrophages will carry latent CMV that may be reactivated through an inflammatory reaction, a prominent feature of COVID-19. Macrophages will likely be activated by SARS-CoV-2 itself, by oxygen radicals, nitric oxide, Toll-like receptor (TLR) stimulation, and cytokines to produce IL-1, IL-6, TNF-α, and GM-CSF. In COVID-19, the most prominent are IL-6, IL-10, and TNF-α. The CMV immediately-early gene promoter contains NF-KB responsive elements that are activated by TNF-α, which can stimulate cells to activate CMV replication [50]. Thus, TNF-α can directly stimulate firing of the CMV immediate-early promoter and CMV reactivation. TNF-α levels are elevated in COVID-19 patients, and may provide a direct link to CMV reactivation. SARS-CoV-2 activates macrophages by establishing a vicious cycle of M1 type macrophage polarization that will promote reactivation of latent CMV and fuel further inflammation.

CMV induces COX-2 expression and production of prostaglandin E2 (PGE2) [51], expression of 5-lipoxygenase [52], and production of leukotriene B4, both powerful inflammatory mediators that drive inflammation. The CMV protein US28 regulates COX-2 expression and phosphorylates STAT3, leading to production of vascular endothelial growth factor and IL-6 [53] and induces smooth muscle cell migration linked to cardiovascular diseases [54]. IL-6 is another cytokine that mediates CMV reactivation [55], also prominent in COVID-19. CMV also induces the production of other cytokines and chemokines that can exacerbate inflammation, such as IL-1β, IL-2, IL-8, IL-10, IL-17, G-CSF, GM-CSF, and TNF-α , MCP-1, MIP-1α, and IP10 [16, 56, 57]. These are similar to those produced in COVID-19 patients. Thus, both viruses activate similar immune pathways and CMV may hence contribute to the cytokine storm in COVID-19 patients. This cytokine storm combined by a weakened interferon response seem to contribute to the severe forms of COVID-19 disease [58]. A reactivated CMV infection will also dampen the IFN response[59] that is important in the combat of SARS-CoV-2, making the situation worse. CMV infection also induces immunosuppressive factors such as TGF-β and IL-10 and produces an IL-10 homologue [16, 60]. Specifically, TGF-β may contribute to fibrosis development [61] and is found in the lungs of COVID-19 patients. Furthermore, CMV directly supresses T cells and natural killer cell functions, thereby helping the virus avoid immune-mediated elimination.

Even in mild to moderate COVID-19, there is a precipitous drop in the abundance of CD4 and CD8 T cells, B cells, and natural killer cells. As a result, control of latent CMV decreases, enhancing risk of reactivation and development of clinical CMV disease, as observed in immunosuppressed patients. In blood, there is often a more moderate drop in monocytes, eosinophils, and basophils—the most prominent infiltrating cells in the lungs of COVID-19 patients. Many of the infiltrating macrophages would carry in latent CMV to the lungs that would be reactivated by the ongoing inflammation. D-dimer and fibrinogen levels are usually high, and there is evidence that microthrombi formation is prominent in the lungs of COVID-19 patients [62]. The coagulopathy is also associated with enhanced risk for stroke and myocardial infarction, which may be first symptoms of COVID-19 in young people. CMV is linked to deep vein thrombosis, stroke, and myocardial infarction, and CMV-infected endothelial cells trigger microthrombi formation *in vitro*[63]. Thus, two life-threatening components of COVID-19—overactivation of the immune system and a coagulopathy—are both are linked to CMV, which in theory should be reactivated in a substantial proportion of COVID-19 patients.

If CMV causes a vicious cycle to drive inflammation and immunosuppression, at which stage does CMV enter this cycle? Is the immune system first suppressed to allow for establishment of a chronic CMV infection, which could further impair the immune response to other infections? Or, will a reactivated CMV infection cause hyper inflammation and simultaneous immunosuppression and lead to high risk of severe COVID-19 in a more acute phase, due to their similarities in immune activation pathways? The question then becomes if this scenario is less likely to occur in CMV negative individuals, who may have a larger naïve T cell pool able to combat SARS-CoV-2, and no reactivation driving inflammation and immunosuppression? Studies referred to indicate that this may be the case, and future studies should therefore search for CMV in COVID-19 patients and address this question in more depth.

The diagnosis of CMV is easily missed in the ICU, as few doctors are aware of this existing problem and the high risk of CMV reactivation in patients with inflammatory conditions. MIS-C in children could involve CMV as a co-factor, perhaps through unlucky timing of a primary or a reactivated CMV infection and coinfection with SARS-CoV-2. Virus induced immune senescence in the elderly may involve CMV reactivation, and could with increasing age be detrimental. Some reports found CMV in COVID-19 patients. In a study of 38 patients on mechanical ventilation in ICU care, 50 % of them showed evidence of CMV reactivation [64]. An elderly patient was reported to be co-infected with SARS-CoV-2 and CMV died after 5 days in the ICU (10.12890/2020_001652). In one case report, CMV DNA was detected in nasopharangeal swabs by sequencing—an unlikely finding in an otherwise healthy individual (10.1101/2020.03.05.20032011). CMV reactivation, colitis and hypovolemic shock was observed in a critically ill patient undergoing experimental treatment for COVID-19. [65]. In studies of T cell activation to CMV peptides *in vitro*, there was a clear trend for enhanced activation of CD8 positive T cells from COVID-19 patients as compared with T cells from controls [66–68], but too few individuals were studied to demonstrate a statistical significance. A recent study used a machine learning based method to predict the risk of COVID-19 severity in 4510 adults and found that CMV specific antibodies were the strongest predictors of infection risk [69]. Patients hospitalized by COVID-19 also showed greater antibody responses to individual CMV and HSV-1 peptides, than those who were not hospitalized [70]. The hospitalized patients also had a higher incidence of both CMV and HSV- infections, but they had a less strong antibody response. In contrast, antibody responses to peptides from common cold viruses such as Rhinoviruses, Influenza viruses and Enteroviruses were higher in patients not hospitalized due to COVID-19 [70]. Although these observations suggest a higher activity of CMV in COVID-19 patients, especially in those who were hospitalized and in need of ICU care, these interpretations should be made with caution as the cohorts were small in size (101 hospitalized and 131 non-hospitalized patients) and data interpretations may be influenced by demographic factors. The hospitalized patients had higher age, were more often men, and of non-white ethnicity. These are known risk factors for both CMV and COVID-19. Of particular relevance is the age dependent weaker immune response that was observed in this study, and as described above, a well-known phenomenon in the elderly. A weakened immune response would be expected to reduce the control of CMV; and CMV activity was indeed higher among the hospitalized, who were older. This weakened immune response may also impact on an individual’s ability to respond to vaccinations. Some studies suggest that CMV positive individuals respond less efficiently to vaccinations [71, 72], which is a concerning factor for the ongoing pandemic. If so, this may affect the efficacy of the ongoing world-wide COVID-19 mass vaccinations of elderly people.

Clinical diagnostics may be challenging in the early stages of local CMV reactivation (e.g., in the lung or bowel). CMV colitis may be diagnosed in tissue biopsy specimens in patients without CMV viremia. Bronchial lavage is a superior method to identify CMV reactivation in the lungs by immunohistochemical staining of infected cells, but is not always feasible. It may be possible to detect CMV IgM, and viremia tests by PCR may or may not identify the virus in its early phase. CMV will not be found unless we look for it. If this virus is reactivated in COVID-19 patients, anti-CMV treatment could potentially reduce inflammatory overload—a possibility that merits prompt consideration and underscore the importance of CMV testing in COVID-19 patients. Methods for CMV testing should include testing for CMV IgM, and preferably examination of a tissue sample by immunostaining for CMV, or testing of a respiratory or bowel sample for CMV by PCR, as PCR may fail to detect its target in blood.

## Conclusions

In conclusion, CMV reactivation and virus induced immune dysfunction may be underestimated as a driver of immunopathogenesis in patients with severe COVID-19. Therefore, studies should be undertaken to investigate the possibility that CMV reactivation sometimes drives the inflammatory response often persisting in patients long after SARS-CoV-2 is no longer detectable in the patient, and may hence be relevant also for patients with long COVID who still have symptoms three months after they were infected [73]. As antiviral drugs are available that may lower the viral activity and inflammatory response, diagnosing CMV in COVID-19 patients could be well worth the effort.

## Data Availability

Not applicable.
